# Sulfated polysaccharides of some seaweeds exhibit neuroprotection via mitigation of oxidative stress, cholinergic dysfunction and inhibition of Zn – induced neuronal damage in HT-22 cells

**DOI:** 10.1186/s12906-020-03047-7

**Published:** 2020-08-14

**Authors:** Tosin A. Olasehinde, Ademola O. Olaniran, Anthony I. Okoh

**Affiliations:** 1 Applied and Environmental Microbiology Research Group (AEMREG), Department of Biochemistry and Microbiology, University of Fort Hare, Alice, Eastern Cape 5700 South Africa; 2 SAMRC Microbial Water Quality Monitoring Centre, University of Fort Hare, Alice, Eastern Cape 5700 South Africa; 3 Nutrition and Toxicology Division, Department of Food Technology, Federal Institute of Industrial Research Oshodi, Lagos, Nigeria; 4 Discipline of Microbiology, School of Life Sciences, College of Agriculture, Engineering and Science, University of Kwazulu-Natal, Durban, South Africa

**Keywords:** Alzheimer’s disease, Neurodegeneration, Sulfated polysaccharides, Seaweeds, Neuroprotection

## Abstract

**Background:**

Sulfated polysaccharides from marine algae are known to possess antioxidative activities, however, their therapeutic role in metal-induced neurodegeneration has not been explored. In this study, the neuroprotective potentials of sulfated polysaccharides isolated from *Ecklonia maxima* (PKPM), *Gelidium pristoides* (PMNP), *Ulva lactuca* (PULV), *Ulva rigida* (PURL) and *Gracilaria gracilis* (PGCL) against Zn-induced neurodegeneration in rats’ hippocampal neuronal cells (HT-22) were assessed.

**Methods:**

Cells were cultured and maintained at 37 °C. Control cells did not contain Zinc sulphate (ZnSO_4_) while other experimental groups contain Zn (50 μM) alone or in combination with sulfated polysaccharides (0.4 or 0.8 mg/mL). Cell viability was assessed using MTT assay while apoptotic assay was also determined using acridine orange and ethidium bromide staining technique. Oxidative stress parameters (superoxide dismutase and catalase activities, glutathione and nitric oxide levels) and acetylcholinesterase activity were also assessed in neuronal cells treated with or without Zn.

**Results:**

Zn significantly reduced cell viability to about 50%. However, sulfated polysaccharides improved cell viability to about 95%. The sulfated polysaccharides also prevented late apoptosis and necrosis triggered by Zn. Furthermore, superoxide dismutase and catalase activities including glutathione content were significantly low in cells induced with Zn. Treatment with sulfated polysaccharides triggered a significant increase in antioxidant enzymes and glutathione content as well as a decrease in the activity of acetylcholinesterase in cells treated with Zn.

**Conclusion:**

PKPM, PGCL, PURL, PULV and PMNP exhibit neuroprotective effects against neuronal damage induced by Zn and this may be attributed to inhibition of apoptosis, oxidative damage and acetylcholinesterase activity. These polysaccharides may be good therapeutic agents to protect neuronal cells against Zn - induced pathological processes associated with Alzheimer’s disease.

## Background

Alzheimer’s disease (AD) is one of the most common devastating neurodegenerative disorder which occurs mostly in elderly individuals. It is characterized by progressive memory decline, behavioural dysfunction, and learning problems [1]. Over 46 million individuals have been diagnosed with AD and this is expected to increase to about 74.7 million by 2030 [2]. Neuropathological processes such as beta-amyloid aggregation, cholinergic dysfunction, oxidative stress-induced neurodegeneration are believed to contribute to the development and progression of AD [3]. Some metals including iron, zinc, and copper play physiological roles in brain function, however, accumulation of these metals in the neurons has been identified as one of the pathological processes involved in the development of AD [4, 5]. Elevated levels of metal ions have been shown to initiate oxidative damage to neurons and contribute to synaptic dysfunction and neurodegeneration which are manifested in AD [4, 6, 7]. Previous report has also shown that Zn ions are present in the hippocampal region of the brain and mediate spatial and memory learning, however, its elevated levels contribute to disruption in amyloid precursor processing pathway and induce amyloid-beta production and aggregation [8]. Cholinesterase inhibitors are commonly used as a therapeutic strategy for the management of AD. Antioxidants have also shown to be effective in mitigating oxidative stress-induced neuronal damage in AD [9, 10]. Sulfated polysaccharides have been identified as antioxidants due to their metal chelating and radical scavenging activities [11]. Some of these polysaccharides are referred to as fucoidans, ulvans, and laminarans depending on the source of macroalgae. Algal polysaccharides are used as nutraceuticals, functional foods, cosmeceuticals, and novel drugs [11].

Some algal polysaccharides contain monosaccharides and sulfate groups which contribute to their biological activities. Some of the biological activities exerted by sulfated polysaccharides isolated from marine algae include immunomodulatory, anticancer, antiviral, anti-allergic, anticoagulant, antidiabetic, and antioxidant effects [12, 13]. The neuroprotective effects of sulfated polysaccharides have not been fully explored, although some reports have shown the therapeutic effects of fucoidans and laminarans against beta-amyloid-induced neurotoxicity in different experimental models [14, 15]. The neuroprotective effects of fucoidans against hydrogen peroxide-induced oxidative stress in neuronal cells have also been reported [16]. However, there is paucity of reports on the effect of algal polysaccharides against metal-induced neuronal damage.

In this study, the neuroprotective potentials of sulfated polysaccharides from species of brown algae (*Ecklonia maxima*), red algae (*Gracilaria gracilis* and *Gelidium pristoides*) and green algae (*Ulva lactuca* and *Ulva rigida*) were investigated in hippocampal neuronal (HT-22) cells induced with Zn.

## Methods

### Materials

Griess reagent and 3-(4,5-dimethylthiazol-2-yl)-2,5-diphenyltetrazolium bromide (MTT) Fetal bovine serum (FBS), epinephrine, trichloroacetic acid, Acetylcholine iodide, 5,5*′*-dithiobisnitrobenzoic acid (DTNB) and phosphate-buffered saline (PBS) were obtained from Sigma Aldrich (St Louis, USA). Zinc sulfate was sourced from Merck (Germany).

### Identification of algal species

*Gelidium pristoides* and *Ulva rigida* were collected from marine environment in Port Alfred, Eastern Cape, South Africa. *Ulva lactuca* and *Gracilaria gracilis* were sourced from Wild Coast Abalone, East London South Africa while Kelp (Pty), South Africa provided *Ecklonia maxima* as reported in our previous studies [17, 18]. Dr. Paul-Pierre Steyn identified all the algal species and voucher specimens were deposited in the herbarium at the Department of Botany, Nelson Mandela University, South Africa.

### Extraction of sulfated polysaccharides

The extraction of the sulfated polysaccharides from *E. maxima* (PKPM), *G. pristoides* (PMNP), *U. rigida* (PURL), *U lactuca* (PULV) and *G. gracilis* (PGCL) was done as previously described [19, 20]. Each seaweed was dried at 25 °C and was ground into powder using a blender, after which they were de-pigmented in different flasks with 400 mL of hexane. After 24 h, the hexane was removed. After drying off the solvent, 400 mL of water was added to flasks containing each seaweeds (200 g) and was heated for 2 h at 90 – 95 °C. The liquid was removed and centrifuged at 4000 g at 25 °C for 5 min. The supernatant was allowed to cool and precipitated with ethanol. The mixture was left overnight and polysaccharides formed were removed by centrifugation (4000 g at 25 °C for 2 min). The residues (polysaccharides) were lyophilized using a freeze dryer (CHRIST Alpha 1–2 LD plus, Germany**)**. The dried samples were stored in vials at 4 °C and used for further analysis.

### Cell culture experiment

HT-22 cells (hippocampal neuron cell line) were provided by Prof Dave Schubert at Salk Institute for Biological Sciences, California, USA. Cells were grown in medium containing dubelcco’s modified eagle medium, FBS (10%), penicillin (2%, U/mL) with streptomycin 100 μg/mL and were kept in CO_2_ (5%) incubator set at 37 °C. After the cells had grown to 60–70% confluence, they were trypsinized and plated in 96 or 24 well plates depending on the experiment. The cells were plated into the following groups: Control (without treatment); Zn: cells treated with 50 μM of ZnSO_4_; PKMP: cells treated with 0.4 or 0.8 mg/mL of sulfated polysaccharide (SP) from *E. maxima* and Zn (50 μM), PGCL: cells treated with 0.4 or 0.8 mg/mL SP from *G. gracilis* and Zn (50 μM); PMNP**:** cells treated with 0.4 or 0.8 mg/mL SP from *G. pristoides* and Zn (50 μM); PULV**:** cells treated with 0.4 or 0.8 mg/mL SP from *U. lactuca* and Zn 50 (μM) and PURL: cells treated with 0.4 or 0.8 mg/mL SP from *U. rigida* and Zn 50 (μM). After treatment with Zn and/or SP, cells were incubated for 18 h. For Biochemical assays, cells were harvested, lysed, and placed on ice for further experiments [21].

### MTT assay

Percentage cell viability was determined in cells treated with Zn and/or sulfated polysaccharides in neuronal cells using MTT assay. A hundred microliters of cells were plated in 96-well plate at 37 °C in a humidified atmosphere at 5% CO_2_ and were treated appropriately with Zn or sulfated polysaccharides after 24 h. Treatment was done for 18 h after which the medium was removed from each well and cells were rinsed with PBS. After this, medium (100 μL) and 20 μL MTT (1 mg/mL) were added to the wells. After incubation for 4 h at 37 °C, the mixture in each well was removed and dimethyl sulfoxide was added. The absorbance of the solution formed was recorded at 570 nm and percentage viability was calculated.

### Determination of apoptosis

Neuronal apoptosis was determined in untreated cells as well as those treated with polysaccharides and/or Zn using ethidium bromide and acridine orange dual stains. After cells were seeded in 24 well plates in appropriate conditions, they were treated with Zn and/or polysaccharides as indicated in the groupings above in triplicates. Treated and untreated (control) cells in 24 well plates were placed in CO_2_ (5%) incubator set at 37 **°**C. The medium was removed from the wells after 18 h and each well was rinsed with PBS. Cells in each well were stained with a mixture of ethidium bromide (100 mg/mL) and acridine orange (100 mg/mL) (1:1 v/v). Fluorescent micrographs of cells in each well were obtained using an Olympus fluorescence microscope equipped with a CC12 fluorescent camera.

### Determination of catalase activity

Catalase (CAT) activity was determined by adding 20 μL of homogenized cells to 50 mM sodium phosphate buffer (pH 7.0, 240 μL) and 2 M H_2_O_2_ (100 μL). The absorbance of the solution was measured at 240 nm within 3 min at an interval of 1 min. Catalase activity was measured as μmoles H_2_O_2_ consumed per milligram protein [22].

### Determination of superoxide dismutase (SOD) activity

Cell homogenates were added to a carbonate buffer (200 μL) after which adrenaline (17 μL) was added to the solution. The absorbance of the solution was measured at 570 nm for 2 min within 15 min [23]. The SOD activity was expressed as percentage inhibition.

### Estimation of glutathione content

Trichloroacetic acid (10%) was added to the cell homogenates to achieve deproteinization. The solution obtained was centrifuged for 5 min at a speed of 3500 rpm. The supernatant (100 μL) obtained was removed and placed in 96 well plates followed by the addition of 50 μL of DTNB. After 5 min, the absorbance of the yellow colour formed was measured at 415 nm [24]. A standard curve was obtained to measure the concentration of GSH.

### Assay of nitric oxide levels

One hundred microliters of the samples were mixed with 100 μL of Griess reagent and a mixture of vanadium chloride (200 μL, 0.2%) and HCl (5%). The solution was incubated for 1 h at 37 °C and absorbance was read at 548 nm. NO produced was measured through the reduction of nitrate to nitrite by vanadium chloride. Nitrite produced was measured as micromol. Per milligram protein.

### Determination of acetylcholinesterase activity assay

Cell homogenates were added to a mixture of phosphate buffer (0.1 M, 150 μL, pH 7.8) and DTNB (6.6 mM, 50 μL). Acetylcholine iodide (6.6 mM, 50 μL) was added to the mixture and the absorbance of a yellow solution formed was measured at 412 nm at 2 min interval. The activity of the enzyme was measured as micromoles AChE per min per milligram of protein [25].

### Statistical analysis

Data were expressed as mean ± standard deviation (SD). One-way analysis of variance and post hoc Tukey*’*s test was used for statistical analysis and means at *p* < 0.05 were significantly different. Graphpad Prism 5.0 was used for the statistical analysis.

## Results

### Effects of treatment with sulfated polysaccharides and Zn on cell viability

**Fig. 1:**
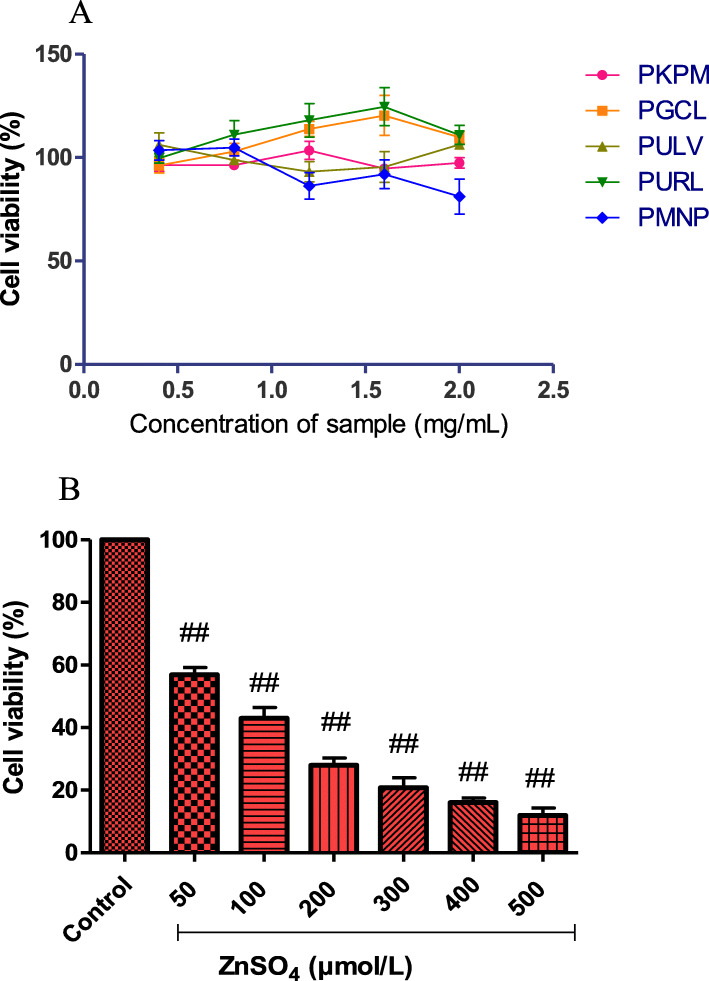
Effect of Sulfated polysaccharides (SP) (**a**) and Zinc sulfate (**b**) on cell viability

Treatment with different concentrations of sulfated polysaccharides (PKPM, PGCL, PULV, PURL, and PMNP) did not cause any observable toxic effects on the neuronal cells (Fig. 1). The result obtained from the MTT assay revealed that none of the sulfated polysaccharides showed cell viability below 95% at concentrations of 0.4 and 0.8 mg/mL. PKPM, PGCL, PULV, and PURL increased cell viability which revealed that they improved the growth of the cells (Fig. 1a). However, treatment with varying concentrations of Zn ranging from 50 to 500 μM caused noticeable toxic effects on the cells as shown in Fig. 1b. The concentration (50 μM) causing about 50% cell viability was used for subsequent experiments.

**Fig. 2:**
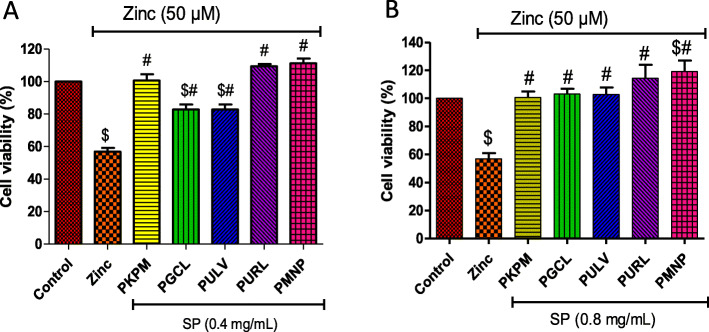
Protective effect of sulfated polysaccharides against zinc-induced neuronal damage in HT-22 cells

Figure 2 depicts the effects of the sulfated polysaccharides on cell viability in the presence of 50 μM of Zn. Treatment with the sulfated polysaccharides (0.4 and 0.8 mg/mL) significantly reduced Zn – induced cytotoxicity in HT-22 cells as shown in Fig. 2a and b. At 0.4 mg/mL, PURL, PMNP, and PKPM showed a significant reduction in cytotoxicity compared to PGCL and PULV. Moreover, an increase in the concentration of the polysaccharides to 0.8 mg/mL increased cell viability and there was no significant difference compared to the control as shown in Fig. 2b. The sulfated polysaccharides significantly improved cell viability at 0.8 mg/mL compared to 0.4 mg/mL. Hence, this concentration was used in subsequent experiments.

### Effect of polysaccharides on apoptosis in Zn –induced neuronal cells

**Fig. 3:**
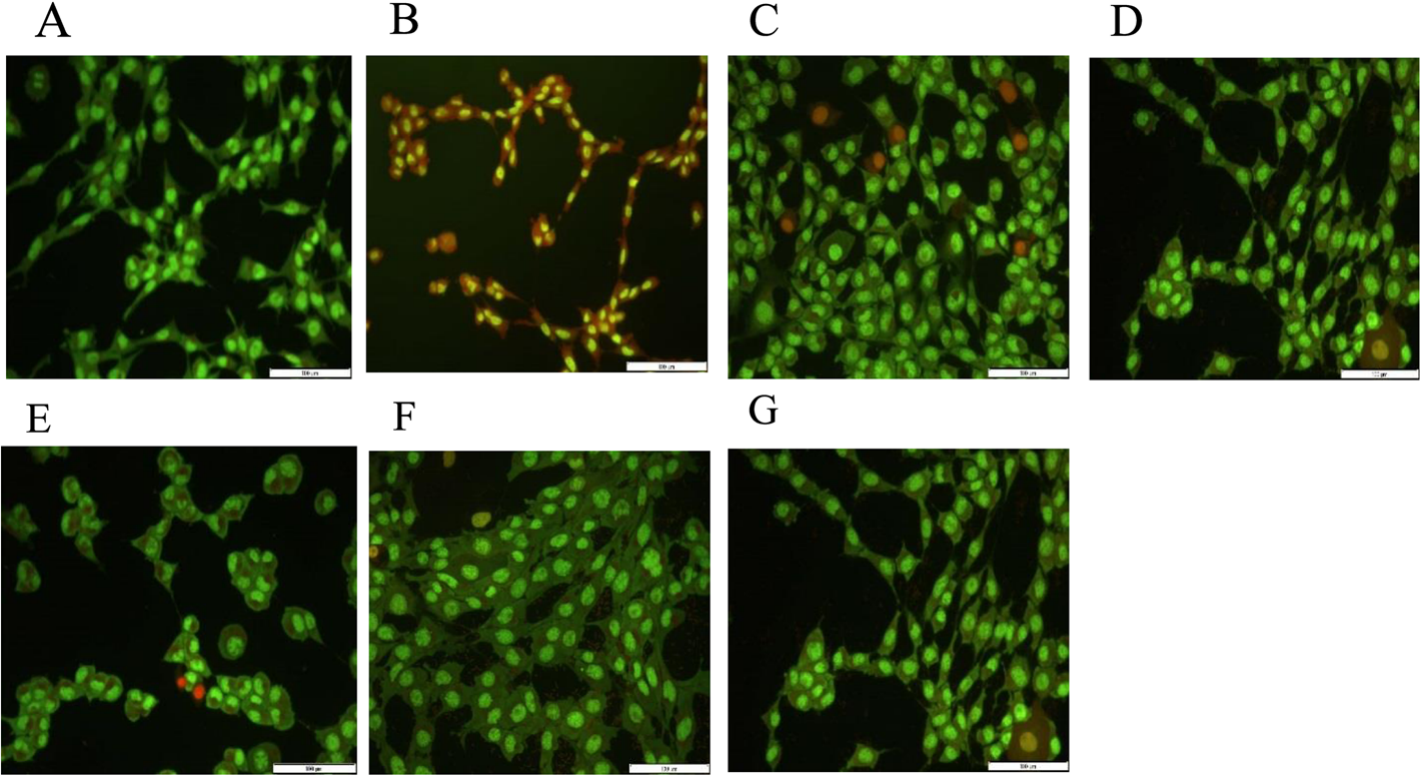
Representative fluorescent micrographs of dual acridine orange and ethidium bromide stained cells revealing morphological changes in HT-22 cells

Figure 3 shows representative fluorescent micrographs of cells treated with Zn (50 μM) and/or sulfated polysaccharides (0.8 mg/mL). The cells were stained with acridine orange and ethidium bromide. There were little or no observable apoptotic cells in the control as shown in Fig. 3a. An increase in late apoptotic and necrotic cells was observed in cells treated with Zn alone as revealed by Fig. 3b. However, after treatment with the sulfated polysaccharides, a decrease in late apoptotic and necrotic cells was observed (Fig. 3c – g). An increase in the number of viable cells was observed in cells treated with PKPM (Fig. 3c) compared to the control (Fig. 3a).

### Effect of sulfated polysaccharides on oxidative stress parameters

**Fig. 4:**
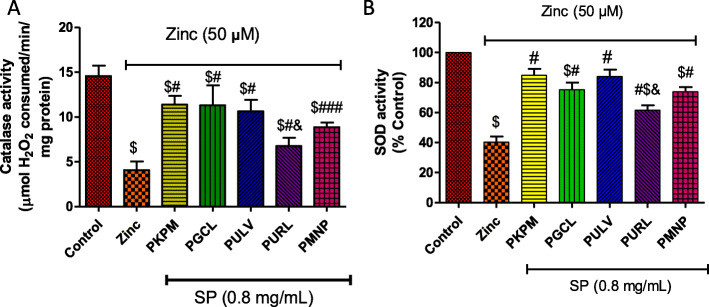
Effect of treatment with sulfated polysaccharides (SP) on (**a**) catalase and (**b**) superoxide dismutase (SOD) activities in Zn-induced neuronal damage in HT-22 cells

Zn (50 μM) significantly reduced catalase activity in the neuronal cells as shown in Fig. 4a However, the sulfated polysaccharides (0.8 mg/mL) increased levels of catalase activity. No significant difference was observed amongst PKPM, PGCL, and PULV, however, these sulfated polysaccharides increased catalase activity in HT-22 cells treated with Zn compared to PURL and PMNP. Similarly, the sulfated polysaccharides increased SOD activity in cells treated with Zn as depicted in Fig. 4b. Treatment with PKPM and PULV showed a significant increase in SOD activity compared to other polysaccharides but were not significantly (*P* > 0.05) different from the control.

**Fig. 5:**
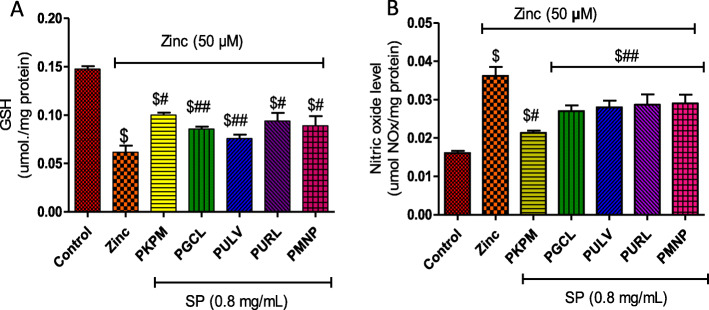
Effect of treatment with sulfated polysaccharides (SP) on (**a**) glutathione and (**b**) nitric oxide levels in Zn-induced neuronal damage in HT-22 cells

Similar results were obtained in the assessment of glutathione content after the cells were induced with Zn (50 μM), the level of the non-protein thiol significantly reduced as shown in Fig. 5a. Treatment with Zn (50 μM) also increased NO levels compared to the control and cells treated with sulfated polysaccharides. as shown in Fig. 5b. NO levels were significantly reduced after treatment with the sulfated polysaccharides (Fig. 5b). Moreover, treatment with PKPM caused a significant decrease compared to other polysaccharides.

### Effect of sulfated polysaccharides on acetylcholinesterase

**Fig. 6:**
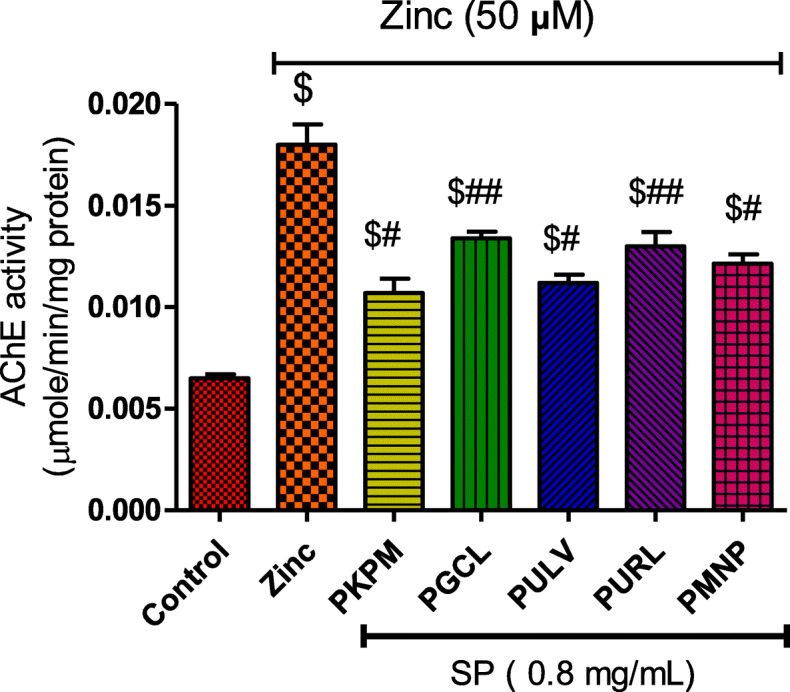
Effect of treatment with sulfated polysaccharides (SP) on acetylcholinesterase activities in Zn-induced neuronal damage in HT-22 cells

Higher acetylcholinesterase activity was observed in cells treated with Zn (50 μM) alone (Fig. 6). After, treatment with the sulfated polysaccharides, acetylcholinesterase activity was significantly reduced in the cells but was higher than the control (Fig. 6). PKPM, PULV, and PMNP significantly reduced acetylcholinesterase activity in the cells compared to PGCL and PURL.

## Discussion

In this study, the effect of some algal polysaccharides on Zn-induced neuronal damage in hippocampal cells was investigated. These polysaccharides have been fully characterized in our previous study and were tentatively identified as fucoidan (PKPM), ulvans (PULV and PULT), and carrageenan (PGCL and PMNP) [19, 20]. The presence of sulfate moiety was confirmed in these polysaccharides including monosaccharides such as fucose, rhamnose, glucose, mannose, galactose, xylose, and allose. The results of this study revealed that Zn exhibited cytotoxic effects at concentrations ranging from 50 to 500 μM. Impairment in homeostasis or accumulation of biologically functional metal in the brain has been associated with the pathogenesis of Alzheimer’s disease [4]. Although Zn plays some neurophysiological functions in the brain, the elevated intracellular concentration of this metal ion can induce neuronal death which has been linked with cholinergic dysfunction, memory loss, and learning problems in AD [26]. Furthermore, Berry and Toms [27] reported that hippocampal neuronal cells are susceptible to cell death induced by Zn. Previous reports have also shown that fucoidans can mitigate H_2_O_2_-induced neurotoxicity in neuronal cells [15, 16]. However, this is the first report on the protective effects of sulfated polysaccharides from *G. gracilis, U. lactuca, G. pristoides*, *E. maxima,* and *U. rigida,* against Zn-induced neuronal death in HT-22 cells. The sulfated polysaccharides improved cell viability in Zn –treated cells. It was observed that PKPM and PMNP enhanced the growth of the cells compared to other polysaccharides. Results from the apoptotic experiment showed that the sulfated polysaccharides inhibited apoptosis in Zn - treated cells. Acridine orange and ethidium bromide dual staining technique is a sensitive method for the detection of apoptotic cells. Ethidium bromide selectively stains non-viable cells and emits yellow to red fluorescence while acridine orange permeates all the cells and emits green fluorescence [28]. The representative fluorescent micrograph of cells treated with Zn (50 μM) revealed condensed chromatin yellow/orange cells which suggest late-apoptotic cells. Some necrotic cells were also observed after treatment with Zn alone. This result suggests that treatment with Zn induced marked apoptotic cell death in HT-22 cells compared to the control and sulfated polysaccharides. However, treatment with the sulfated polysaccharides did not show necrotic cells, although late apoptotic cells were observed in cells treated with PULV. Early apoptotic cells were also observed in cells treated with PGCL and PURL. A significant increase in the number of viable cells was also observed after treatment with PKMP as shown in Fig. 3c. This is an indication that the sulfated polysaccharides used in this study exhibited neuroprotective effects against Zn-induced neuronal death and the mechanism involved may be via inhibition of apoptotic pathways. Previous experimental investigation has shown that fucoidan inhibits apoptosis induced by hydrogen peroxide in PC-12 cells via reduction of caspase-3 expression and activation of AKT phosphorylation [29].

There are indications that the accumulation of metals induces neurodegeneration in the brain by triggering the production of free radicals which attacks neuronal cells [7, 30]. In this study, treatment with Zn caused a decrease in SOD and CAT activities as well as low glutathione levels which may lead to the generation of free radicals and in turn trigger oxidative injury in neuronal cells. Antioxidant enzymes function as a defense mechanism against oxidative stress in cells. Moreover, levels of these enzymes are low in the neurons and this makes them more susceptible to oxidative damage [31]. The susceptibility of the neuronal cells to free radical attack may contribute to the decrease in SOD and CAT activities observed in cells treated with Zn. Furthermore, the high content of polyunsaturated fatty acids, mitochondrial dysfunction, and increase in unfavourable space and volume ratio of microglial cells may give rise to the production of superoxide radicals which are capable of attacking the neurons, hence causing neuronal damage [32]. Accumulation of reactive oxygen species in neurons may trigger deleterious intracellular responses ultimately caused by oxidative stress [33]. In this study, exposure of the neuronal cells to Zn disrupted the antioxidant defense mechanisms, hence causing oxidative damage to neurons. This result is consistent with the apoptotic cell death which was observed after treatment with Zn. However, treatment with PKPM, PGCL, PULV, PURL, and PMNP significantly increased superoxide dismutase and catalase activities as well as glutathione content which suggests an improvement in antioxidant status of the neuronal cells treated with Zn. This result agrees with the findings of [29] which showed that fucoidan induced the activation of antioxidant enzymes in H_2_O_2_ – induced apoptosis in PC12 cells. Although knowledge on the mechanism of action of the polysaccharides is limited, their neuroprotective effects could be linked to their antioxidant activity which can be associated with their sulfate content, radical and metal chelating activities. Some studies have also attributed the antioxidant properties of sulfated polysaccharides to low molecular weight and sulfate content [34, 35].

Oxidative stress precedes inflammation, hence, there is a link between metal-induced oxidative stress and neuroinflammation [36]. Production of nitric oxide via the activation of inflammatory signals in the brain has been associated with neuroinflammation which is an important pathological process involved in AD [37]. NO is known to induce both oxidative and nitrosative stress which induces neuronal apoptosis via activation of p55 MAPK pathway, activation of mitochondrial permeability transition, and endoplasmic reticulum stress [36]. In this study, Zn induced an increase in the production of NO in HT-22 cells compared to the control group. The observed elevated levels of NO in Zn- treated cells may contribute to neuronal loss and/or death. This is consistent with the necrotic and late apoptotic cells observed in the fluorescent micrographs of Zn-treated cells. Evidence has shown that the overproduction of NO is associated with neuronal loss, nerve injury, and protein aggregation in AD [38, 39]. However, PKPM, PGCL, PMNP, PULV, and PURL significantly reduced NO levels in Zn-induced neuronal cells. This result suggests that these polysaccharides may possess anti-inflammatory potentials against Zn-induced neuroinflammation in HT-22 cells. This result also correlates with the investigation carried out by Park et al. [40] which revealed that fucoidan inhibits NO production in lipopolysaccharide-induced BV-2 microglial cells.

The cholinergic pathway is important for the transmission of nerve impulses and memory function. Moreover, acetylcholinesterase plays a significant role in the cholinergic system as a regulatory enzyme that controls the levels of acetylcholine – an important neurotransmitter required for neurotransmission [41]. However, cholinergic dysfunction or disruption in the cholinergic pathway has been identified as a pathological process in the development of AD [42]. The use of acetylcholinesterase inhibitors has proven to be an effective therapeutic approach involved in alleviating cholinergic dysfunction in AD. In this study, the activity of acetylcholinesterase was determined in cells treated with Zn and/or combination with sulfated polysaccharides. Cells treated with Zn alone exhibited high acetylcholinesterase activity which indicates an alteration in cholinergic function. However, treatment with the sulfated polysaccharides reduced acetylcholinesterase activity in Zn – treated rats. This result revealed that PKPM, PGCL, PMNP, PURL, and PULV may ameliorate cholinergic deficit caused by metal-induced neurotoxicity. This result correlates with findings in previous studies as Park et al. [43] reported that sulfated polysaccharide isolated from *Ecklonia cava* showed competitive and non-competitive inhibitory effects on acetylcholinesterase in PC12 cells induced with H_2_O_2_. Gao et al. [44] also reported that fucoidan improved cognitive function in beta-amyloid-induced memory impairment in rats’ brain via modulation of acetylcholine levels and acetylcholinesterase activity. The inhibitory effect of sulfated polysaccharides on acetylcholinesterase has been attributed to their ability to bind effectively to the anionic site which lies in the gorge of the enzyme thereby reducing its activity [43]. Hence, sulfated polysaccharides could be potential neuroprotective agents capable of mitigating cholinergic deficit in Alzheimer-like pathological conditions.

## Conclusion

This study revealed that PKPM, PGCL, PULV, PURL and PMNP exhibit neuroprotective potentials via their antioxidative properties, capacity to inhibit neuronal apoptosis, and ability to improve cholinergic function in Zn-induced neuronal damage in hippocampal neuronal cells. Furthermore, the protective potentials of the sulfated polysaccharides against oxidative stress-induced neuronal damage and inhibition of acetylcholinesterase suggest that they are good antioxidants with the potentials to prevent neurodegeneration, cholinergic dysfunction, and neuroinflammation in pathological conditions associated with AD. However, the neuroprotective mechanism of these polysaccharides needs to be investigated further using different experimental models.

## Data Availability

The data sets used and/or analyzed in this study will be made available from the corresponding author on reasonable request.

## Abbreviations

AD: Alzheimer’s disease
CAT: Catalase
DTNB: 5,5*′*-dithiobisnitrobenzoic acid
FBS: Fetal bovine serum
GSH: Glutathione
HT-22: Neuronal hippocampal cells
MTT: 3-(4,5-dimethylthiazol-2-yl)-2,5-diphenyltetrazolium bromide (MTT)
NO: Nitric oxide
PGCL: Sulfated polysaccharides from *G. gracilis*
PKMP: Sulfated polysaccharide from *E. maxima*
PMNP: Sulfated polysaccharide from *G. pristoides*
PULV: Sulfated polysaccharides from *U. lactuca*
PURL: Sulfated polysaccharides from *U. rigida*
SOD: Superoxide dismutase
SP: Sulfated polysaccharide
Zn: Zinc
